# When hunger and thirst part ways: a longitudinal comparison of men and women across the Ramadan fasting-recovery cycle, with non-fasting controls

**DOI:** 10.3389/fnut.2026.1859780

**Published:** 2026-09-18

**Authors:** Chen Fleischmann, Mahmood Sindiani, Maria Korman

**Affiliations:** 1 Occupational Therapy Department, Temporal Organization of Behavior (ToBe) Laboratory, Ariel University, Ariel, Israel; 2 The Levinsky-Wingate Academic College, Wingate Institute, Netanya, Israel

**Keywords:** appetite regulation, circadian rhythm, drinking behavior, longitudinal studies, religious intermittent dry fasting, sex, thirst regulation

## Abstract

**Introduction:**

The Ramadan fast imposes abstinence from food and water during daytime for 28–29 days, providing an ecological model of prolonged intermittent dry fasting. Longitudinal data characterizing hunger, thirst, and hydration trajectories across fasting and recovery remain limited.

**Methods:**

In the 2021–2023 Circadian Ramadan (CiRAM) Study (registered in the Open Science Framework, DOI: https://doi.org/10.17605/OSF.IO/ETMU4), self-reported daily peak hunger and thirst levels, daily peak appetite timing, and estimated daily fluid intake were assessed in a prospective longitudinal cohort of 287 Ramadan-observing Israeli Muslims (mean age 26.89 ± 6.77, 58.2% female) and 76 ethnically comparable controls (mean age 24.28 ± 6.55, 63.2% female) at five time points spanning pre-, early-, and late-fasting and two post-fasting timepoints.

**Results:**

Hunger declined significantly at Ramadan onset and remained suppressed one month post-Ramadan (medium effect, η^2^ = 0.067), with a more rapid within-sex decline in women (η^2^ = 0.098 vs. 0.038). The sexes differed significantly only at one-month recovery, when women reported higher hunger (medium effect, Cramér’s V = 0.21). Thirst rose incrementally, remained elevated through late fasting, then fell below pre-Ramadan baseline after fasting (medium effect, η^2^ = 0.071). Peak appetite timing was delayed during Ramadan and returned to baseline post-Ramadan (small effect, η^2^ = 0.052). Fluid intake rose post-Ramadan, earlier in men (small effect, η^2^ = 0.04). Normalized intake was higher in women throughout in the Muslim group and at 3 timepoints in controls. Controls showed no comparable transition-specific changes, though a small omnibus reduction in hunger was observed (small effect, η^2^ = 0.051).

**Discussion:**

Over Ramadan and recovery, partially dissociable trajectories were observed: progressive hunger suppression contrasted with an incremental rise in thirst, alongside a circadian delay in peak appetite timing. Sex differences were limited to hunger and fluid intake. The absence of comparable changes in controls suggests these trajectories are not attributable to seasonal variation alone, with effects on hunger, thirst, and fluid intake persisting one month after Ramadan.

## Introduction

Fasting, defined as abstinence from food and, in the case of “dry fasting,” also from fluids (1), is a state of temporary deprivation, eliciting compensatory physiological responses through hunger and thirst (2, 3). Despite both driving food and fluid intake to restore homeostasis, hunger and thirst are fundamentally distinct processes with differing activation patterns, regulatory mechanisms, and urgency of behavioral response (2, 3). As subjective, conscious, physiological state perceptions, hunger and thirst display substantial individual variability (4, 5). Unlike directly measurable physiological parameters, both are assessed by self-reporting or effector-triggered behavior such as eating and drinking, despite weak correlations with hunger and thirst (6–8).

Thirst signals an immediate threat, and fluid deprivation can impair physiological and cognitive function more rapidly than food abstinence (3, 9–13). The development of hunger and thirst during fasting and recovery differs between individuals, reflecting hormonal, metabolic, physiologic, and behavioral variation (14, 15). With advancing age, hunger and thirst intensity decline due to reduced neuroendocrine and osmoreceptor sensitivity (16, 17). Men and women respond differently to fasting due to dimorphism in the metabolism of energy substrates such as glucose and lipids (18), and a sex-specific brain response to hunger and satiety (19). Body composition may also play a role in the development of hunger, as a higher body fat percentage has been linked with higher self-reported hunger before eating (20), while regular physical activity may reduce hunger intensity and limit dehydration-related thirst (21, 22). These physiological adaptations in the timing, frequency, and intensity of hunger and thirst should therefore be regarded in a population-specific context, yet how sex and age modulate them during prolonged dry fasting remains uncharacterized.

An observational chronobiological study conducted in participants’ natural environment during the month of Ramadan can serve as an ecological manipulation, representing a self-administered perturbation of nutritional and social routines, unlike laboratory-based interventions, that are typically short-term and lack real-world validity. During Ramadan, food and fluid intake follow a well-established set of temporal rules that align fasting and eating with distinct phases of the circadian cycle.

Over 1.5 billion practicing Muslims worldwide are estimated to observe the month-long Ramadan fast each year, also known as Ramadan diurnal intermittent fasting (RDIF) (1, 23). The fast is temporally defined by sunrise and sunset, requiring partial reversal of the sleep-eat/drink cycle, so that food and drink are abstained during daylight and permitted during the dark hours (24, 25). During this period, daytime work/study activities are maintained, though often delayed and shortened, alongside more frequent religious, family, and community-oriented practices, while habitual physical activity and energy expenditure are often reduced (25–28). Ramadan fasting is held in the highest regard, and considered an obligation for healthy adult Muslims, not otherwise exempt for age, health or travel related reasons (26). Set by the 354-to-355-day Islamic (Hijri) calendar, Ramadan shifts 10–11 days earlier each Gregorian year, completing a full seasonal cycle in approximately 33 years (29). The resulting seasonality shift, along with geographical latitude and day length, may considerably impact the fasting experience, particularly in hotter climates (29).

The temporal trajectories of hunger and thirst across different phases during Ramadan remain uncharacterized, as existing studies predominantly employ endpoint comparisons (pre- vs. post-Ramadan) or single-timepoint assessments, failing to capture dynamics across early fasting, late fasting, and recovery. To address this gap, this longitudinal controlled study examined daily peak hunger and thirst levels, daily peak appetite timing, and fluid intake across five time points spanning pre-Ramadan baseline through early and late fasting phases and into early and late recovery, with comparison to non-observing controls to isolate fasting-specific effects.

Specifically, the research questions (Ramadan-observing group) were:

a) Is there an effect of the Ramadan fast on the daily peak hunger, thirst levels, daily timing of peak appetite, and fluid intake?
b) Are there sex-specific effects on the time course of hunger and thirst levels?
c) Can the observed changes be attributed to the Ramadan fast or to other non-Ramadan-specific effects, such as seasonality?

## Methods

The present investigation was conducted as part of the CiRAM (Circadian Ramadan) Study, or internet-based Circadian Ramadan study (Registration DOI: https://doi.org/10.17605/OSF.IO/ETMU4). The study was ethically approved by the Ariel University Human Research Ethics Committee of the Faculty of Health Sciences (AU-HEA-MK-202110316-1). All participants were informed of the objectives of the study as well as the identities of the principal and coordinating investigators. Prior to the initiation of the experimental procedures, electronic informed consent was obtained, wherein participants agreed to take part in the survey and to the collection of personal contact information to facilitate repeated assessments over time.

### Study design and data collection

The study was carried out in two study groups: fasting Muslims and non-Muslim controls recruited from Arabic non-Muslim communities. The study examined the effect of Ramadan fast on the time-course of hunger level and timing and thirst levels along with reported fluid intake patterns. Data collection timepoints were spaced to allow at least seven days of adaptation following major transitions (onset of fasting, end of fasting) before assessment, enabling physiological adjustment to perturbations in sleep–wake and eating-fasting daily cycles (see Figure 1). The time axis presents the days when the personal link to the survey at each study phase was sent. Participants were requested to complete the survey within 48 h of receiving the link (a reminder was sent at 24 h), with more than 90% completed within 24 h at each phase.

**Figure 1:**
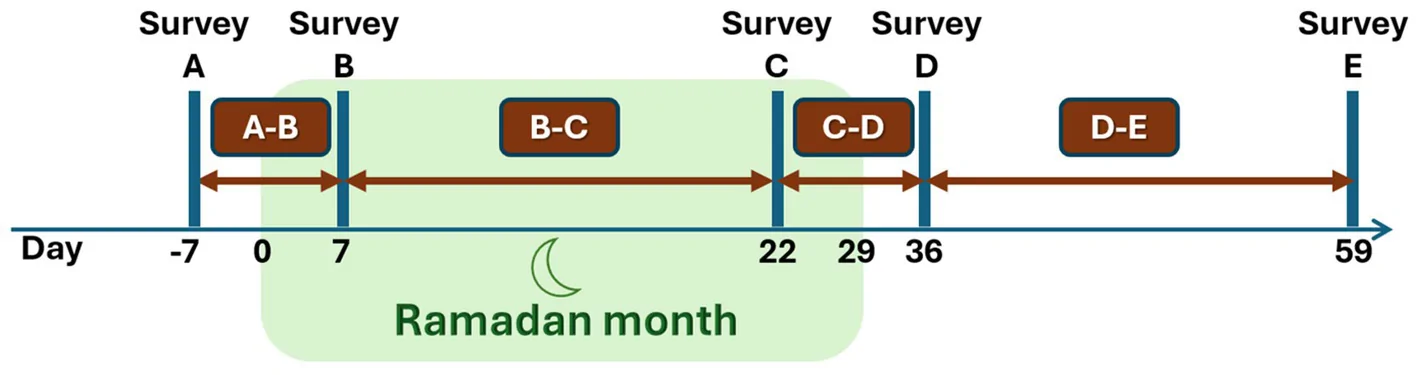
The schematic representation of the data collection timeline, days relative to the first day of Ramadan. Each participant completed five surveys: At point A (baseline) – one week before the beginning of the Ramadan fast, point B (early fasting phase) – at the end of the first week of the fast, point C (late fasting phase) – during the fourth week of Ramadan, point D (early recovery phase) – at one week after the end of Ramadan, and point E (late recovery phase) – at four weeks after the end of Ramadan.

Recruitment was conducted through digital advertisements distributed across university channels, academic and non-academic social networks, as well as email-based outreach. Prospective participants completed a brief electronic screening questionnaire that included basic sociodemographic questions regarding age, health status, and shift work, to verify eligibility based on the study’s inclusion criteria. Data collection took place during the Ramadan fasts of 2021, 2022 and 2023 via the QualtricsXM online survey platform (Qualtrics Survey Software; Provo, Utah, US). Each participant contributed data during a single Ramadan year (2021, 2022 or 2023, springtime). Participants completed the 5 surveys via handheld device or computer, each requiring approximately 15–20 min to complete. Participants answered the survey in Arabic. The questionnaires were structured across multiple pages, and responses were reviewed for internal consistency prior to data storage. In instances of illogical or inconsistent input, an on-screen prompt notified the respondent of the error and requested correction before submission.

### Participants

A total of 450 adults from Israeli Muslim Arabic-speaking communities and 112 ethnically, culturally and geographically comparable non-Muslim adults from other Arabic-speaking communities (Christian, Druze or no denomination), expressing interest in participation in the study, completed a brief screening questionnaire. Non-Muslim participants responded to a similar set of 5 questionnaires delivered at the same time points during 2023, with Ramadan-specific phrasing removed (e.g., ‘during the last week’ rather than ‘during the first week of Ramadan’).

Of these, 417 Muslim and 101 non-Muslim individuals met the following inclusion criteria: (1) age ≥ 18 years; (2) habitual fasting during the month of Ramadan and the intention to fast during the study period (upcoming Ramadan, Muslim group); (3) healthy in general, no diagnosed sleep, neurological or psychological disorders; (4) no regular medication use (except contraceptives); (5) not shift workers; and (6) women: not pregnant or nursing mothers. Respondents with missing or invalid data during the data-collection period, as well as those self-reporting a “bad” health status or serious illness, were excluded from the analysis. Two hundred eighty-seven Muslim and 76 non-Muslim respondents aged 18–59, were included in the analytical sample (Figure 2). Participants were residing in multiple regions in Israel (Northern area, Jerusalem, Central area, Southern areas). Supplementary material SM-1, provides the detailed description of the number of participants per year, attrition and compliance rates.

**Figure 2:**
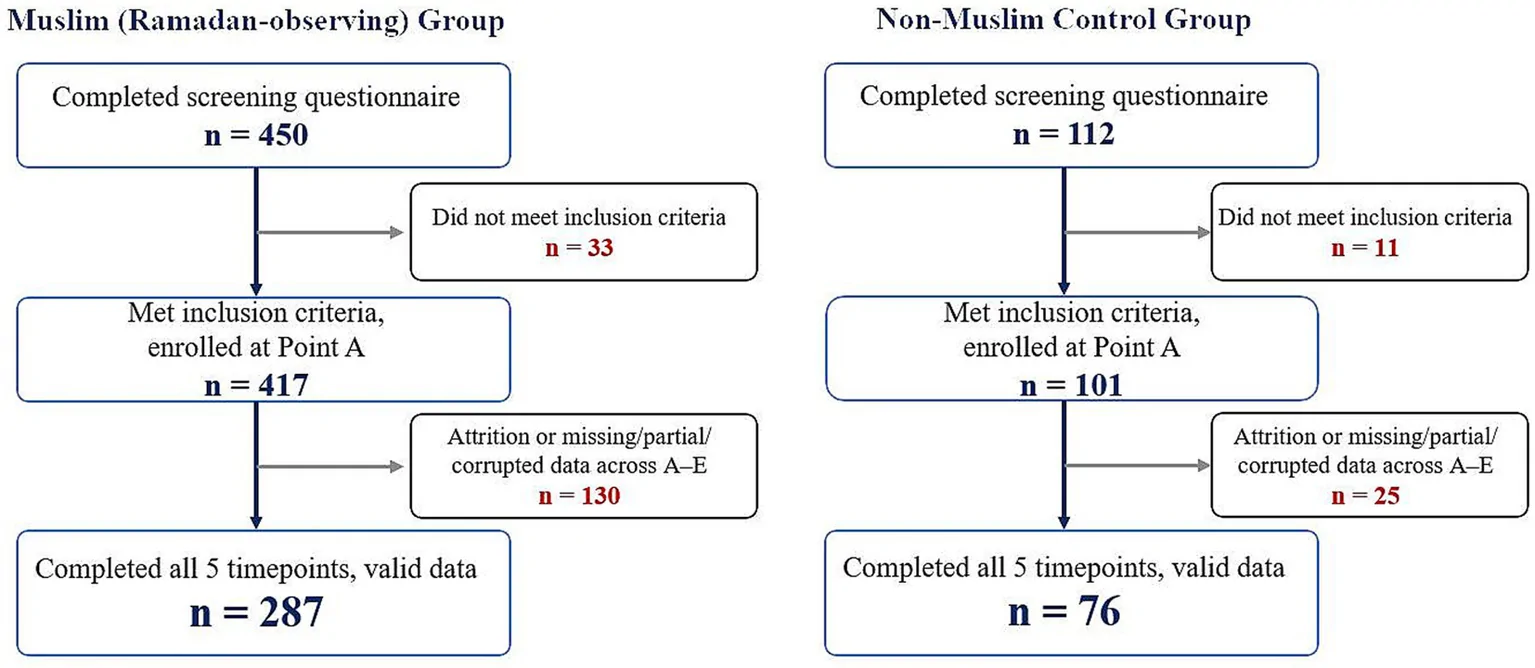
STROBE diagram of the participant screening, eligibility, and attrition process for the Muslim (Ramadan-observing) and non-Muslim control groups. Numbers reflect participants completing the initial screening questionnaire, meeting inclusion criteria, and completing all five study timepoints (A–E) with valid data. Full year-by-year detail is provided in Supplementary material SM-1.

### Measures

Sociodemographic variables were assessed at study phase A: these included age, self-reported sex (male/female; hereafter referred to as sex), education level, marital status, satisfaction with financial level, employment, lifestyle and general health. Changes in body weight during Ramadan were reported at point E compared to point A with the questions: “Do you think that changes occurred to your weight during the month of Ramadan? (Yes/No)” and “My weight after Ramadan compared with my weight before Ramadan: change in kilograms.” The reported change in weight was compared with the weight reported at baseline to obtain the percent change in weight for each individual.

### Hunger and thirst levels, daily peak appetite timing and fluid intake assessment

Hunger and thirst levels were assessed separately using the questions: “During the last week, what was your strongest hunger/thirst level during the day?” with the categorical answers: “I never felt hungry/thirsty,” “I felt slightly hungry/thirsty,” “I felt moderately hungry/thirsty,” “I felt very hungry/thirsty”.

The daily timing of peak appetite was assessed with the question: “During the last week, when did you usually feel your strongest appetite during the day?” with the categorical answers: “in the morning hours/around noon/in the afternoon hours/in the evening hours/at night hours/I had an appetite steadily throughout the day.” The change in the daily peak appetite timing was assessed over time by evaluating the direction of change (Earlier, No change, Later) while transitioning between paired study time points A–B, B–C, C–D and D–E. Cases in which a participant reported a constant level of hunger throughout the day in one or more of the paired time points were designated “Steady”.

Daily fluid intake was assessed using the question: “During the last week, how much fluid did you consume on average in 24 h (without physical exertion or unusual physical activity)? Please estimate consumption in the number of 250 mL drinking glasses. Beverage glasses include water, coffee, tea, milk, soda (sweetened drinks).” Ordinal whole numbers ranging from 3 to 20 were accepted as answers. The estimated daily fluid intake is presented both as an absolute value (L/day) and as a body-mass–normalized value (mL/kg/day) , using the reported body weight to account for differences in body size. It should be noted that estimates of daily fluid intake expressed in cups per day inherently exclude fluids derived from food sources (e.g., foods with high water content such as fruit or soups).

### Statistical analyses

All statistical analyses were conducted using IBM SPSS Statistics Version 29.0 (IBM Corp., Armonk, NY). Within-subject non-parametric repeated assessments were performed at time points A, B, C, D and E. Sex-specific and Ramadan-fasting sensitivity analyses vs. the control sample were performed at each time point, over the entire study duration (i.e., A → E) and in the transitions between individual time points (e.g., A → B, see Figure 1). Longitudinal within-subject changes across the five time points (A, B, C, D, E) were evaluated using the non-parametric Friedman test for repeated measures. When the Friedman test indicated significant omnibus effects, pairwise comparisons between specific time points (e.g., A → B) were performed using Wilcoxon signed-rank tests, applying Bonferroni correction for 4 planned pairwise comparisons (A → B, B → C, C → D, D → E, resulting in adjusted *α* = 0.0125 (0.05/4)). Additional exploratory comparisons to baseline/endpoint were evaluated at *p* < 0.05.

To examine sex differences (men vs. women) at each of the five time points (A–E) within the Muslim sample, Pearson’s chi-square tests were employed for ordinal outcomes and Mann–Whitney U tests for continuous variables. As sex differences were examined across five time points, a Bonferroni correction was applied, resulting in an adjusted α = 0.01 (0.05/5). Sex-stratified comparisons were not conducted within the control group due to the insufficient number of participants per sex. Where relevant, Cramér’s V was calculated to estimate the strength of association between sex and categorical outcomes. For non-normally distributed continuous variables (weight change and % weight change), medians and inter-quartile ranges (IQR) were reported.

Pearson’s chi-square tests were also used to compare categorical changes in peak appetite timing (Earlier, No change, Later, Steady) between groups (Muslim vs. Control) and between sexes (male vs. female, within the Muslim sample) across consecutive time-point transitions. For significant overall effects, post-hoc analyses of adjusted standardized residuals were performed to identify the categories contributing most to the observed differences. All statistical tests were two-tailed, and statistical significance was accepted at *p* < 0.05, unless otherwise specified.

Daily peak hunger, thirst, and appetite timing, excluding the ‘steady throughout the day’ category (referred to as “specific daily peak appetite timing”), were analyzed separately in the Muslim fasting group and the non-fasting control group using ordinal generalized linear models with a cumulative logit link and Type III likelihood ratio tests. The models included sex, time point, and age as predictors, with a sex × time interaction term retained. For thirst, the model including the sex × time interaction did not achieve stable convergence, attributable to a sparse cell in the Muslim male “very thirsty” category at baseline. As the sex × time interaction term was non-significant (χ^2^(4) = 0.756, *p* = 0.944), it was removed and the thirst model was re-estimated without it in both the Muslim and control groups. Age was modeled as a continuous covariate. These models did not include a random effect or repeated-measures correlation structure for the within-participant clustering across the five time points, in contrast to the hydration models described below.

Absolute and relative hydration were analyzed with linear mixed-effects models estimated by restricted maximum likelihood, with a random intercept for participant and an AR(1) covariance structure for repeated measures. Fixed effects comprised sex, age, and time point, along with the sex × time interaction. Degrees of freedom were estimated using the Satterthwaite approximation.

Individual time-point contrasts from these models are reported uncorrected for multiple comparisons, as exploratory estimates following a significant omnibus effect.

### Socio-demographic, adherence and environmental characteristics of the sample

No major sex differences were observed in the Muslim group in socio-demographic measures including age, employment, education level, and marital status (see Table 1). Mean age of the Muslim participants was 26.89 ± 6.77, with women consisting 58.2% of the sample. Most Muslim participants were unmarried (65.9%), most of the Muslim sample worked or studied at the time of the survey (89.9%), and the majority held an academic degree (63.8%). Financial satisfaction was high with 91.3% of Muslim participants reporting feeling confident to some degree about their financial status. Sex was significantly associated with self-defined financial status (Pearson’s χ^2^(4, 286) = 9.68, *p* = 0.046, Cramér’s V = 0.18). ASRs did not indicate any over- or under-represented financial status category. More than half of Muslim participants (53%) resided in the north, while over a quarter resided in the Jerusalem area (27.2%), 16.4% in central Israel and only 3.4% in the south of Israel. Over half the Muslim sample (54%) were city dwellers, with the remainder living in villages (Table 1).

**Table 1:** Sociodemographic characteristics of the sample.

Group	Muslim	Muslim women	Muslim Men	Muslim men vs. women *p* value	Control	Control women	Control Men	Control men vs. women *p* value	Muslim vs. control *p* value
*n*, %, statistics	(*n* = 287), %	(*n* = 167), %	(*n* = 120), %	(t-test or χ^2^)	(*n* = 76), %	(*n* = 48), %	(*n* = 28), %	(t-test or χ^2^)	(t-test or χ^2^)
Sex (self-reported)				<0.001				<0.001	ns
Male	41.8				36.8				
Female	58.2				63.2				
Age, mean (SD)	26.89 (6.77)	26.34 (7.17)	27.67 (6.12)	ns	24.28 (6.55)	23.69 (4.88)	25.29 (8.72)	ns	<0.001
Marital status				ns				ns	<0.001
Single\divorced\other	65.9	67.1	64.2		89.5	87.5	92.9		
Married	34.1	32.9	35.8		10.5	12.5	7.14		
Geographic area				ns				ns	<0.001
North	53	53.9	51.7		90.8	93.8	85.7		
Jerusalem (east and west)	27.2	24	31.7		0	0	0		
Centre	16.4	19.2	12.5		9.2	6.3	14.3		
South	3.4	3	4.2		0	0	0		
Residence				ns				ns	ns
City	54	54.5	53.3		61.8	54.2	75		
Village	46	45.5	46.7		38.2	45.8	25		
Education level				ns				ns	0.004
< 12 education years	1.7	1.2	2.5		0	0	0		
12 education years	24.7	26.3	22.5		43.4	37.5	53.6		
Professional without academic degree	9.8	12.6	5.8		13.2	10.4	17.9		
Academic degree or student of higher education	63.8	59.9	69.2		43.4	52.1	28.6		
Self-defined financial status				0.046				ns	ns
Very financially confident	13.6	15.7	10.8		11.8	16.7	3.6		
Happy with financial status	44.1	48.8	37.5		43.4	37.5	53.6		
Moderately confident, short term	33.6	27.7	41.7		32.9	35.4	28.6		
Unsatisfied – does not fully cover basic needs	8	6.6	10		11.8	10.4	14.3		
Extremely unsatisfied – cannot afford basic needs	0.7	1.2	0		0	0	0		
Employment				ns				ns	ns
Working\studying	89.7	86.4	94.2		89.5	89.6	89.3		
Not working\studying	10.3	13.6	5.8		10.5	10.4	10.7		

Mean ± SD or frequency (% of group total), *p*–value: Significance of the differences between groups using t-test, Mann–Whitney U, or χ^2^ as appropriate.

Health and lifestyle characteristics are represented in Table 2. Most participating Muslims (63.5%) reported a traditional religious lifestyle, with 32% defined themselves as religious, to some degree. Health, based on self-reporting, was mainly very good to excellent (91.3%), with most participants (61.7%) reporting a regular exercise routine of at least 30-min sessions, twice a week. Exercise frequency did not differ significantly between Muslim and control participants (χ^2^(4, 361) = 9.42, *p* = 0.051, Cramér’s V = 0.16). Sex was significantly associated with exercise frequency (Pearson’s χ^2^(4, 285) = 17.45, *p* = 0.002, Cramér’s V = 0.25), Adjusted standardized residuals showed men were over-represented among those exercising 4–6 times per week (ASR = 3.65), with women correspondingly under-represented (ASR = −3.65). 27.2% of the Muslim male participants had reported smoking often or regularly, compared to 15.7% of female Muslim participants. Sex was significantly associated with smoking status (Pearson’s χ^2^(4, 286) = 39.99, *p* < 0.001, Cramér’s V = 0.37). Men were significantly over-represented among regular smokers (ASR = 5.01) and underrepresented among those who never smoked (ASR = −5.58), whereas women showed the opposite pattern (regular smokers ASR = −5.01; never smoked ASR = 5.58).

**Table 2:** Health and lifestyle characteristics of the sample.

Group	Muslim	Muslim women	Muslim Men	Muslim men vs. women *P* value	Control	Control Women	Control Men	Control Men vs. Women P value	Muslim vs. Control P value
*n*, %, statistics	(*n* = 287), %	(*n* = 167), %	(*n* = 120), %	(t-test or χ^2^)	(*n* = 76), %	(*n* = 48), %	(*n* = 28), %	(t-test or χ^2^)	(t-test or χ^2^)
Religious lifestyle				ns				ns	< 0.001
Secular, not religious	4.6	6.7	1.7		35.5	33.3	39.3		
Traditional	63.5	66.1	60		51.3	58.3	39.3		
Religious	30.2	26.1	35.8		11.8	6.3	21.4		
Strictly religious	1.8	1.2	2.5		1.3	2.1	0		
Health status				ns				ns	ns
Excellent	48.6	45.8	52.5		43.4	37.5	53.6		
Very good	42.7	44.6	40		38.2	47.9	21.4		
Good	8.4	9.6	6.7		18.4	14.6	25		
Not good	0.3	0	0.8		0	0	0		
Smoking				< 0.001				0.006	ns
I smoke regularly	10.8	3	21.7		9.2	0	25		
I often smoke	16.4	12.7	21.7		13.2	14.6	10.7		
I never smoked	59.1	72.9	40		59.2	68.8	42.9		
I smoked in the past, not currently	5.9	4.8	7.5		3.9	4.2	3.6		
I tried a few times, but now I do not smoke	7.7	6.6	9.2		14.5	12.5	17.9		
Exercise Routine (>30 min)				0.002				ns	0.051
I do not exercise at all	21.4	23.6	18.3		13.2	12.5	14.3		
Once a week	16.8	20.6	11.7		18.4	16.7	21.4		
2–3 times a week	38.9	41.2	35.8		34.2	41.7	21.4		
4–6 times a week	15.1	8.5	24.2		28.9	25	35.7		
Everyday	7.7	6.1	10		5.3	4.2	7.1		
BMI	24.28 (4.04)	23.3 (3.9)	25.6 (3.8)	< 0.001	23.62 (3.75)	22.6 (3.4)	25.3 (3.7)	0.002	ns

Mean ± SD or frequency (% of group total), *p*–value: Significance of the differences between groups using two-tailed t-test or χ^2^test.

Average BMI was 23.3 ± 3.9 in men and 25.6 ± 3.8 in women. Non-Muslim, control group participants presented comparable frequencies in all major socio-demographic, health and lifestyle parameters, apart from relationship status, with a higher rate (89.5%) of unmarried control participants (χ^2^(1, 363) = 16.22, *p* < 0.001, Cramér’s V = 0.21), geographical area, with Muslims most often living in the North (53.0%) and Jerusalem area (27.2%), whereas controls were predominantly located in the North (90.8%) (χ^2^(4, 363) = 39.50, *p* < 0.001, Cramér’s V = 0.33), and religious lifestyle (χ^2^(3, 361) = 61.12, *p* < 0.001, Cramér’s V = 0.41), in which Muslims were significantly less likely to describe themselves as secular (ASR = −7.64) and more likely to describe themselves as religious (ASR = 3.22), whereas control participants showed the opposite pattern (secular ASR = 7.64; religious ASR = −3.22); the traditional category did not differ significantly between groups (ASR = ±1.94) (Tables 1, 2).

Overall, the adherence to a full month of fasting was high. The reported rate of breaking the fast in the week preceding the relevant study points was as follows: 1 male (0.8%), and 7 female (4.2%) participants at point B, and 10 female (6%) participants at point C. Reported reasons were not health related for the male participant and varied for the female participants between menstrual cycle (7 and 6, at phases B & C, respectively), health related (1 and 3, at phases B & C, respectively) and not-health related (1 and 1, at phases B & C, respectively). Data from the Israel Meteorological Service demonstrated that during the 2021–2023 Ramadan months that occurred in spring (March–May), the median daytime temperatures were temperate to warm (mid–high 20s °C, with slight variations across regions). The daylength was ~12–14 h, resulting in a corresponding ~13–15.5 h fasting window, religiously defined from pre-dawn to sunset prayers (30).

Over the Ramadan fast, 64.8% of Muslim participants (67.5% of male and 62.9% of female) answered a non-obligatory question and reported experiencing a change (decrease or increase) in body weight over the Ramadan fast, with no statistical significance observed between sexes (χ^2^(1, 287) = 0.655, *p* = 0.418). When asked to estimate the change in weight, 63.4% of Muslim participants replied. There was a general tendency to report a decrease in weight. No statistical difference was observed between sexes in the reported change in weight: male (*n* = 78; median = −3 Kg, IQR −5 to 2 Kg) vs. female (*n* = 104; median = −2 Kg, IQR −3 to 2 Kg), Mann–Whitney U = 3462.50, z = −1.70, *p* = 0.089 (two-tailed), or in the percent weight change from baseline: male (*n* = 78; median = −3.73%, IQR −5.44 to 2.05%) vs. female (*n* = 104; median = −3.64%, IQR −5.66 to 3.32%), Mann–Whitney U = 3987.0, z = −0.196, *p* = 0.844 (two-tailed).

## Results

### Daily peak hunger level

Across the 5 study points (A–E), there were robust within-subject reductions in daily peak hunger in the Muslim group (Figure 3), as indicated by a Friedman test (χ^2^(4, 281) = 75.831, *p* < 0.001, η^2^ = 0.067), reflecting a medium effect size.

**Figure 3:**
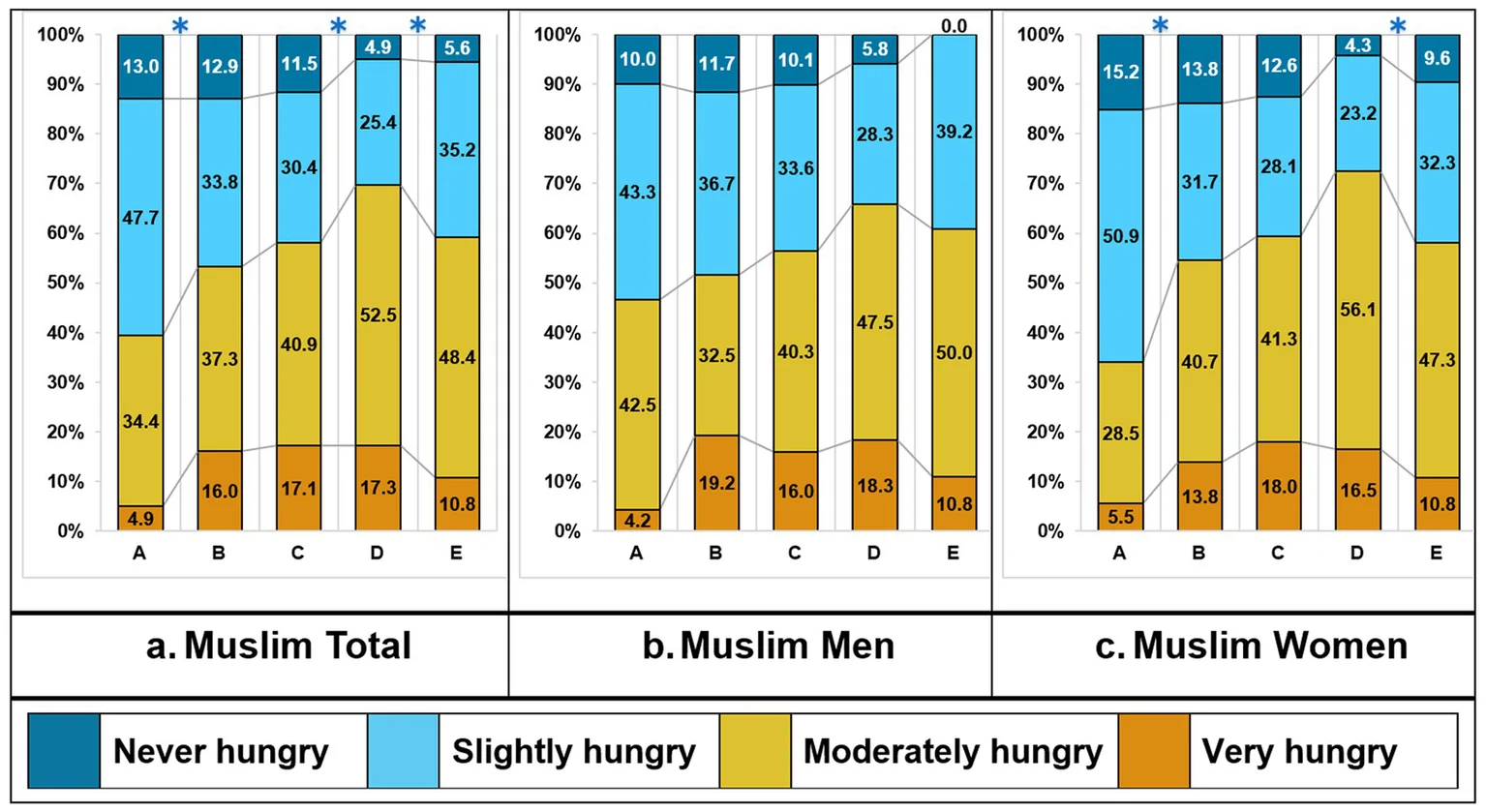
Daily peak hunger level, displayed in percentage of responders, in each study phase (A–E), shown for the total Muslim population **(a)** Muslim men **(b)** and Muslim women **(c)**. Significant planned phase transitions (Bonferroni corrected, *ɑ* = 0.0125), are indicated by an asterisk (*).

Multiple changes in daily peak hunger levels were observed across the 5 study time points (Figure 3; Table 3). At point A, 60.7% of the Muslim sample reported that the daily peak hunger sensation during an average day in the previous week was moderately strong to strong, and the rest reported weaker hunger levels, with no significant differences between sexes (χ^2^(3, 285) = 6.46, *p* = 0.09). At point B (early Ramadan phase), the percentage of participants reporting moderately strong to strong daily peak hunger declined to 46.7% and reached 42.0% by point C (late Ramadan phase). The lowest percentage of participants reporting moderately strong to strong daily peak hunger (30% of Muslims) was observed at point D (early recovery phase). At point E, 40.8% of the sample reported feeling moderately strong to strong daily peak hunger, suggesting that daily peak hunger had not returned to baseline even one month after Ramadan (Figure 3).

**Table 3:** Post hoc Wilcoxon signed-rank test analysis for phase transitions (A–B, B–C, C–D, D–E) for the Muslim sample, of daily peak hunger & thirst levels, appetite timing, and hydration in liters and in mL normalized to body weight (mL/Kg/Day).

Measure	Group	Value	A–B	B–C	C–D	D–E
Daily peak hunger level	Total Muslim	*Z*	**−4.197**	−1.444	**−3.038**	**−3.367**
		*p*	0.000*	0.149	0.002*	0.001*
	Men	*Z*	**−2.021**	−0.348	−1.921	−0.953
		*p*	**0.043**	0.728	0.055	0.34
	Women	*Z*	**−3.781**	−1.594	**−2.343**	**−3.501**
		*p*	0.000*	0.111	**0.019**	0.000*
Daily peak appetite timing	Total Muslim	*Z*	**−3.459**	−1.551	**−3.928**	−0.944
		*p*	< 0.001*	0.121	< 0.001*	0.345
	Male	*Z*	**−3.661**	−0.584	**−2.512**	−0.443
		*p*	0.000*	0.559	0.012*	0.658
	Female	*Z*	−1.41	**−2.633**	**−3.029**	−1.531
		*p*	0.159	0.008*	0.002*	0.126
Daily peak thirst level	Total Muslim	*Z*	**−2.591**	−0.902	**−7.067**	**−2.835**
		*p*	0.010*	0.367	0.000*	0.005*
	Male	*Z*	−1.789	−0.812	**−4.538**	−1.471
		*p*	0.074	0.417	0.000*	0.141
	Female	*Z*	−1.946	−0.499	**−5.423**	**−2.451**
		*p*	0.052	0.618	0.000*	**0.014**
Absolute daily fluid intake	Total Muslim	*Z*	−0.669	−0.683	**−3.061**	**−2.673**
		*p*	0.504	0.495	0.002*	0.008*
	Male	*Z*	−0.049	−0.771	**−2.968**	−0.903
		*p*	0.961	0.441	0.003*	0.366
	Female	*Z*	−0.974	−0.367	−1.407	**−2.543**
		*p*	0.330	0.713	0.159	0.011*
Normalized daily fluid intake	Total Muslim	*Z*	−0.63	−0.725	**−2.95**	**−2.751**
		*p*	0.529	0.468	0.003*	0.006*
	Male	*Z*	−0.081	−0.776	**−2.97**	−0.988
		*p*	0.935	0.438	0.003*	0.323
	Female	*Z*	−0.903	−0.37	−1.3	**−2.595**
		*p*	0.367	0.712	0.194	0.009*

*p* < 0.05 values appear in bold; values also meeting the Bonferroni-corrected threshold (ɑ = 0.0125) are additionally marked with an asterisk (*).

To determine whether hunger declined progressively across fasting phases or showed abrupt transitions, we conducted pairwise within-subject comparisons for transitions between phases A–B, B–C, C–D and D–E using Wilcoxon signed-rank tests. The results confirmed that there were multiple changes across tested transitions (Figure 3; Table 3). Specifically, there were significant reductions in peak hunger levels in pairs A–B and C–D, followed by a significant increase in pair D–E, while, during the Ramadan month itself, the change across the pair B–C was not significant. Strikingly, even after a full month post Ramadan, daily peak hunger levels had not fully returned to baseline in both sexes (pair A–E; Wilcoxon test, Z = −6.023, *p* < 0.001).

Generally, both men and women displayed reductions in daily peak hunger levels across study points A–E (Figure 3) (Friedman test by sex: men, χ^2^(4, 119) = 18.17, *p* = 0.001, η^2^ = 0.038; women, χ^2^(4, 162) = 63.20, *p* < 0.001, η^2^ = 0.098). Only at point E was a significant gap observed between sexes, with women showing higher frequencies of moderate to high hunger in comparison with men (χ^2^(3, 287) = 12.53, *p* = 0.006). Cramér’s V between sex and level of hunger at phase E was V = 0.21 (medium effect size), while no significant associations were found during other phases. Before correction for multiple comparisons, men showed only one significant paired comparison (A–B), while women had three significant changes in paired phase transitions (A–B, C–D, D–E) (Table 3). After Bonferroni correction (*α* = 0.0125), women showed a significant hunger decrease at A → B and a significant increase at D → E, whereas men showed no significant pairwise differences (Table 3). Altogether, these analyses suggest that the time course of both the Ramadan and recovery phases were sex specific.

In the control group, daily peak hunger levels changed significantly across the 5 study points, as indicated by a Friedman test (χ^2^(4, 75) = 15.41, *p* = 0.004, η^2^ = 0.051), with a small effect size. Notably, none of the phase transitions in peak hunger levels of the control participants were significant, consistent with the interpretation that the observed hunger suppression in the Muslim group is unlikely to be explained solely by seasonal or calendar effects (Table 3).

### Daily peak appetite timing

For the fasting Muslim population, the daily timing of peak appetite changed significantly across the 5 study points (A–E), as indicated by a Friedman test (χ^2^(4, 283) = 58.506, *p* < 0.001, η^2^ = 0.052), with a small effect size (Figure 4). *Post hoc* paired comparisons for transitions between points showed that during Ramadan, the timing of peak appetite was delayed compared to point A (Figure 4; Table 3). A full recovery of peak appetite timing was already observed at point D and persisted at point E. Note that at each time point, some participants (6.3–8%) occasionally, and not the same participants, reported having no specific time for peak appetite during the day. Both Muslim men and women displayed significant changes over time in the daily timing of peak appetite (Figure 4), (men: χ^2^(4, 119) = 21.33, *p* < 0.001, η^2^ = 0.045; women: χ^2^(4, 164) = 43.96, *p* < 0.001, η^2^ = 0.067). Post hoc Wilcoxon signed-rank test with Bonferroni correction indicated that among men, the daily timing of peak appetite at point B was significantly later than at point A (Z = −3.66, *p* < 0.001), and at point D (Z = −2.92, *p* = 0.003). Among women, the daily timing of peak appetite was significantly delayed between points B and C (Z = −2.633, *p* = 0.008) and significantly advanced between points C and D (Z = −3.029, *p* = 0.002), with point C representing the latest daily timing for peak appetite, compared to the baseline time point A (Z = −3.156, *p* = 0.002), and 1 month of recovery at point E (Z = −4.951, *p* < 0.001).

**Figure 4:**
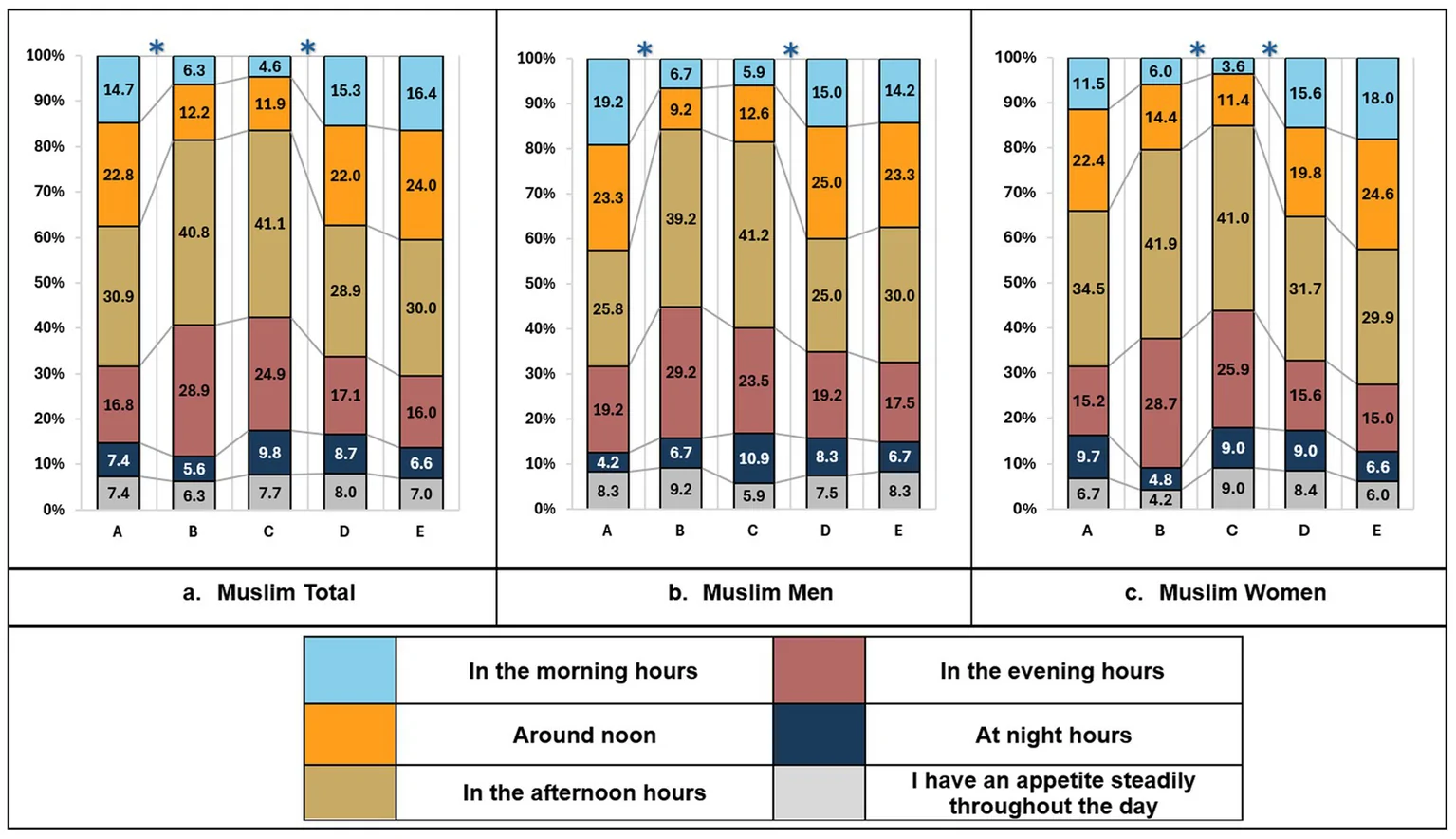
Daily peak appetite timing, displayed in percentage of responders, in each study phase (A–E), shown for the total Muslim population **(a)**, Muslim men **(b)**, and Muslim women **(c)**. Significant planned phase transitions (Bonferroni corrected, ɑ = 0.0125) are indicated by an asterisk (*).

To characterize individual-level changes, we categorized the direction of appetite timing shifts (earlier, no change, later, or steady peak appetite) across consecutive time points (Figure 5a). Peak appetite timing showed bidirectional shifts across Ramadan phases, with the most common pattern being a delay at Ramadan onset (A-B, 40%), relative stability during fasting (B-C, 44.6% no change), an advance immediately post-Ramadan (C-D, 38.3% earlier), and return to baseline thereafter (D-E, 39% no change). In each transition pair, 12.9–14.5% of Muslim participants reported having steady peak appetite throughout the day in at least one time point and were classified as “Steady”.

**Figure 5:**
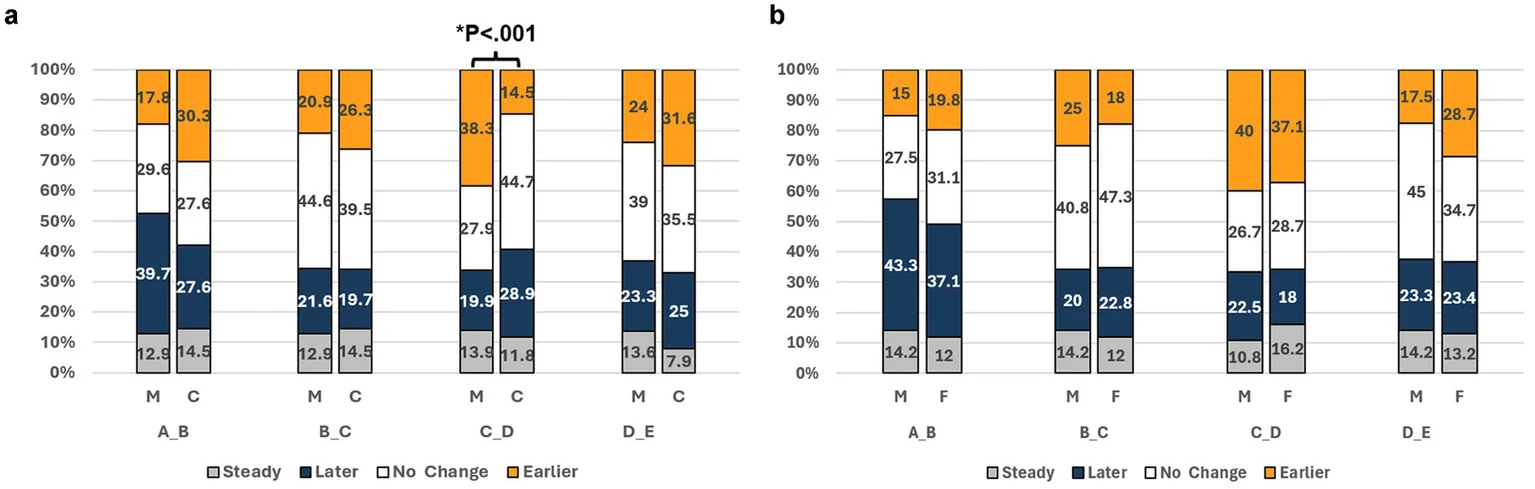
Change in daily peak appetite timing, displayed in percentage of responders, in each study phase (A–E), shown for **(a)** the Muslim and control populations, **(b)** male and female Muslim participants. Significant planned phase transitions (Bonferroni corrected, *ɑ* = 0.0125), are indicated by an asterisk (*). No significant differences were observed between the male and female Muslim sample in the depicted phase transitions (A–B, B–C, C–D, D–E).

No significant difference was observed between male and female Muslims in all paired comparisons of phase transitions, as indicated by Pearson’s chi-square tests (χ^2^(3, 287): A–B = 2.140, *p* = 0.544; B–C = 2.814, *p* = 0.421; C–D = 2.407, *p* = 0.492; D–E = 5.609, *p* = 0.132) (Figure 5b). A detailed description of the peak daily appetite timing analysis during transitions is presented in Supplementary material SM-2.

### Daily peak thirst level

In the Muslim group, changes in daily peak thirst levels showed a biphasic pattern of changes (Figure 6a; Table 3): an increase in thirst during the first week of Ramadan and a rebound decrease immediately post-Ramadan. In particular, at baseline (point A), 48.4% reported moderately strong to strong thirst. This increased significantly to 57.5% at point B and remained stable at point C (53.5%). A dramatic decrease occurred at point D (30.3%), with low thirst levels persisting through point E (37.3%). Altogether, this pattern indicates that even one month post-Ramadan, thirst levels had not fully recovered to baseline levels, with significantly fewer participants reporting moderately strong to strong thirst at point E compared to baseline (A–E: Z = −2.40, *p* = 0.016). In transitions A-B, B-C and C-D, no sex differences were observed in daily peak thirst level changes.

**Figure 6:**
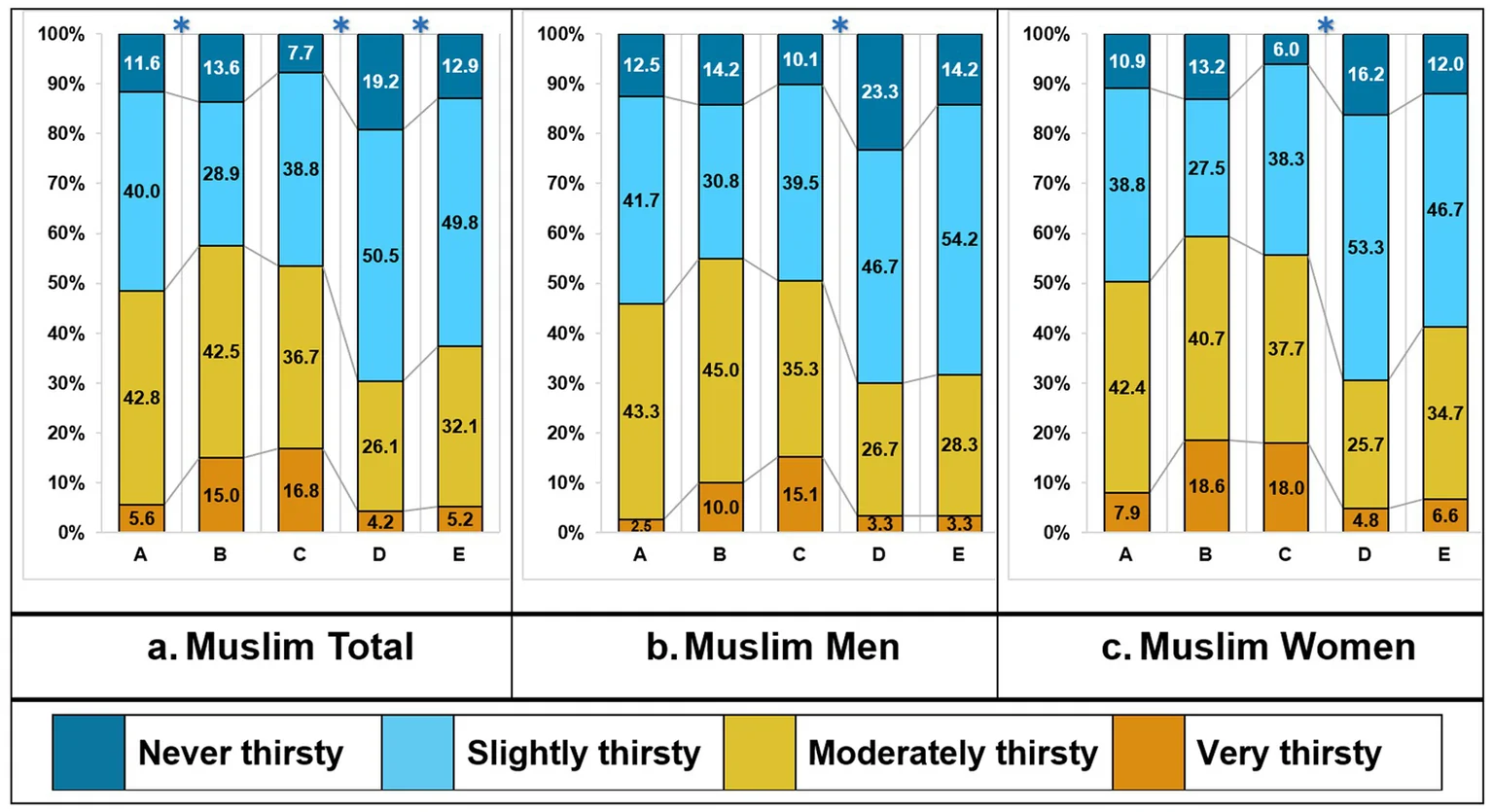
Daily peak thirst level, displayed in percentage of responders, in each study phase (A–E), shown for the total Muslim population **(a)**, Muslim men **(b)**, and Muslim women **(c)**. Significant planned phase transitions (Bonferroni corrected, *ɑ* = 0.0125, for four comparisons), are indicated by an asterisk (*).

During late recovery (D → E transition), women showed a significant trend toward further thirst reduction (Z = −2.451, *p* = 0.014) while men did not (Z = −1.471, *p* = 0.141). Both sexes remained below pre-Ramadan baseline thirst levels at one month post-Ramadan (Figures 6b,c). The Friedman test across time points A–E confirmed an overall significant within-subject decrease in daily peak thirst levels across the study period (χ^2^(4, 284) = 80.865, *p* < 0.001, η^2^ = 0.071), with a medium effect size. The general time course of changes in daily peak thirst levels was independent of sex, with men and women displaying similar changes in daily peak thirst levels across study phases (A–E) (men: χ^2^(4, 119) = 38.49, *p* < 0.001, η^2^ = 0.081; women: χ^2^(4, 165) = 43.24, *p* < 0.001, η^2^ = 0.066).

### Absolute and normalized daily fluid intake

At baseline (time point A), mean absolute daily fluid intake (reported as the daily number of 250 mL cups consumed and expressed in liters per day) in the Muslim sample was 2.33 ± 1.06 Liters/day. Daily fluid intake was maintained during the month of Ramadan at time point B (2.30 ± 1.07 Liters/day) and at time point C (2.29 ± 1.03 Liters/day), and after Ramadan increased to 2.43 ± 1.06 Liters/day at time point D and 2.53 ± 1.04 Liters/day at time point E (see Supplementary Figure SM-3). The Friedman test across time points A–E confirmed an overall significant within-subject increase in daily fluid intake (χ^2^(4, 284) = 39.92, *p* < 0.001, η^2^ = 0.04). *Post hoc* Wilcoxon signed-rank tests revealed that absolute daily fluid intake rose significantly in transitions C-D (Z = −3.061, *p* = 0.002) and D-E (Z = −2.673, *p* = 0.008). An exploratory comparison further showed that fluid intake at time point E was significantly higher than at baseline (time point A) (Z = −3.330, *p* < 0.001). Mann–Whitney U tests of daily fluid intake were used to assess differences between sexes at each time point (Table 4). At time point D, men reported higher absolute daily fluid intake than women (men: 2.57 ± 1.02 Liters/day; women: 2.33 ± 1.09 Liters/day; Z = −2.205, *p* = 0.027); this difference did not reach the Bonferroni-corrected threshold (*α* = 0.01). No significant sex difference in absolute fluid intake was observed at any other time point.

**Table 4:** Mean ± St. Dev. of daily fluid intake in absolute (liters/day) and normalized (mL/kg/day) values, in the total Muslim, male and female Muslim populations.

	Liters/day Mean ± St. Dev					mL/Kg/day Mean ± St. Dev				
	Total Muslim	Men	Women	Sex comparison (Mann–Whitney U test)		Total Muslim	Men	Women	Sex comparison (Mann–Whitney U test)	
*n*	284–287	119–120	165–167	z	*p*	284–287	119–120	165–167	z	*p*
**A**	2.33 ± 1.06	2.36 ± 1.01	2.31 ± 1.09	−0.567	0.571	34.19 ± 16.65	29.61 ± 12.85	**37.52 ± 18.28**	***−3.644***	< 0.001*
**B**	2.30 ± 1.07	2.42 ± 1.11	2.22 ± 1.03	−1.283	0.200	33.81 ± 16.85	30.38 ± 14.23	**36.28 ± 18.14**	***−2.602***	0.009*
**C**	2.29 ± 1.03	2.33 ± 1.00	2.26 ± 1.04	−0.627	0.531	33.49 ± 16.07	29.29 ± 12.82	**36.49 ± 17.46**	***−3.394***	< 0.001*
**D**	2.43 ± 1.06	**2.57 ± 1.02**	2.33 ± 1.09	***−2.205***	***0.027***	35.30 ± 16.00	32.29 ± 13.31	**37.47 ± 17.41**	***−2.430***	***0.015***
**E**	2.53 ± 1.04	2.60 ± 1.06	2.47 ± 1.02	−0.957	0.338	36.91 ± 16.02	32.82 ± 14.01	**39.85 ± 16.76**	***−3.619***	< 0.001*

*p* < 0.05 values appear in bold; values also meeting the Bonferroni-corrected threshold (ɑ = 0.0125) are additionally marked with an asterisk (*). Ramadan time points (B & C) appear in light green.

Next, we performed the same analysis when daily fluid intake was normalized to each individual’s reported body weight (mL/Kg/day). At baseline (time point A), the normalized mean daily fluid intake was 34.19 ± 16.65 mL/Kg/day. Fluid intake during Ramadan was maintained at 33.81 ± 16.85 and 33.49 ± 16.07 mL/Kg/day at time points B and C, respectively. One week after Ramadan (point D), normalized daily fluid intake increased to 35.30 ± 16.00 mL/Kg/day and reached 36.91 ± 16.02 mL/Kg/day at time point E (see Supplementary Figure SM-3). Mean normalized fluid intake for the total Muslim, male, and female Muslim populations is reported in Table 4. Women consumed more fluid per Kg body weight than men across all time points, with significant sex differences surviving Bonferroni correction (ɑ = 0.0125) at time points A, B, C, and E (all *p* ≤ 0.009), but not at time point D (*p* = 0.015). A sex difference in normalized fluid intake was also observed in the control group, reaching significance at time points A, C, and E (see Supplementary material SM-3 for the control group fluid intake table).

The Friedman test in the Muslim sample across time points A–E confirmed an overall significant within-subject increase in normalized daily fluid intake (χ^2^(4, 284) = 39.92, *p* < 0.001, η^2^ = 0.04). *Post hoc* Wilcoxon signed-rank tests demonstrated that normalized daily fluid intake was maintained during Ramadan at levels similar to baseline, with no significant differences in the A-B or B-C transitions (Table 3). Only after the end of Ramadan did normalized daily fluid intake show a significant rebound in the C-D (Z = −2.95, *p* = 0.003) and D-E (Z = −2.751, *p* = 0.006) transitions. The Friedman test across time points A–E confirmed an overall significant within-subject increase in normalized daily fluid intake for both men (χ^2^(4, 119) = 22.88, *p* < 0.001, η^2^ = 0.05) and women (χ^2^(4, 165) = 19.98, *p* < 0.001, η^2^ = 0.03). However, in men, a significant increase in normalized daily fluid intake was seen during the first week of recovery (C-D; Z = −2.97, *p* = 0.003) and when comparing point E with point A (Z = −2.9, *p* = 0.004), whereas women showed gradual increases across both recovery phases (C-D and D-E), indicating more sustained compensation.

### Model-based results

Hunger, thirst, specific appetite timing, and hydration trajectories were modeled separately for the Muslim and control groups, as described in Statistical Analyses. Control group results are presented in the Supplementary material SM-4. Corresponding odds ratios (for hunger, thirst, and appetite timing) and B coefficients (for hydration), each with 95% confidence intervals, are provided in Table 5.

**Table 5:** Ordinal generalized linear model and linear mixed-model results for daily peak hunger, thirst, appetite timing, and hydration in Ramadan-observing Muslim participants.

Outcome	Predictor	Statistic	*p*	OR/B [95% CI]
Peak daily hunger level	Model (omnibus)	χ^2^(10) = 105.52	<0.001	—
	Time (with A as Baseline)	χ^2^(4) = 47.51	<0.001	—
	B vs. A	χ^2^(1) = 15.01	<0.001	0.44 [0.29, 0.67]
	C vs. A	χ^2^(1) = 22.16	<0.001	0.36 [0.23, 0.55]
	D vs. A	χ^2^(1) = 43.44	<0.001	0.24 [0.16, 0.37]
	E vs. A	χ^2^(1) = 17.86	<0.001	0.41 [0.27, 0.62]
	Sex	χ^2^(1) = 0.60	0.439	0.71 [0.46, 1.12]
	Age	χ^2^(1) = 49.61	<0.001	0.95 [0.93, 0.96]
	Sex × Time	χ^2^(4) = 5.93	0.204	n.s.
Peak daily thirst level	Model (omnibus)	χ^2^(6) = 67.15	<0.001	—
	Time (with A as Baseline)	χ^2^(4) = 58.19	<0.001	—
	B vs. A	χ^2^(1) = 5.41	0.020	1.46 [1.06, 2.01]
	C vs. A	χ^2^(1) = 8.50	0.004	1.61 [1.17, 2.21]
	D vs. A	χ^2^(1) = 12.83	<0.001	0.56 [0.41, 0.77]
	E vs. A	χ^2^(1) = 3.19	0.074	0.75 [0.55, 1.03]
	Sex^a^	χ^2^(1) = 2.50	0.114	0.85 [0.69, 1.04]
	Age	χ^2^(1) = 6.53	0.011	0.98 [0.97, 1.00]
Specific peak daily appetite timing	Model (omnibus)	χ^2^(10) = 77.29	<0.001	—
	Time (with A as Baseline)	χ^2^(4) = 56.94	<0.001	—
	B vs. A	χ^2^(1) = 5.15	0.023	1.58 [1.06, 2.35]
	C vs. A	χ^2^(1) = 10.77	0.001	1.96 [1.31, 2.94]
	D vs. A	χ^2^(1) = 0.30	0.584	0.89 [0.59, 1.35]
	E vs. A	χ^2^(1) = 3.03	0.082	0.70 [0.46, 1.05]
	Sex	χ^2^(1) = 0.01	0.935	0.71 [0.45, 1.11]
	Age	χ^2^(1) = 16.38	<0.001	0.97 [0.96, 0.98]
	Sex × Time	χ^2^(4) = 4.20	0.379	n.s.
Absolute hydration	Time (with A as Baseline)	F(4, 661.52) = 5.80	<0.001	—
	B vs. A	t(841.32) = −0.94	0.348	−0.068 [−0.210, 0.074]
	C vs. A	t(926.57) = 0.32	0.753	0.024 [−0.128, 0.177]
	D vs. A	t(794.68) = 0.47	0.637	0.037 [−0.116, 0.189]
	E vs. A	t(747.36) = 2.52	0.012	0.194 [0.043, 0.346]
	Sex	F(1, 282.03) = 1.51	0.221	0.067 [−0.185, 0.319]
	Age	F(1, 281.09) = 1.47	0.226	0.009 [−0.006, 0.024]
	Sex × Time	F(4, 661.53) = 1.52	0.196	n.s.
Relative hydration	Time (with A as Baseline)	F(4, 656.22) = 5.02	0.001	—
	B vs. A	t(837.53) = −0.69	0.493	−0.733 [−2.832, 1.366]
	C vs. A	t(923.73) = 0.34	0.733	0.391 [−1.859, 2.640]
	D vs. A	t(792.71) = 0.37	0.711	0.426 [−1.830, 2.682]
	E vs. A	t(746.11) = 2.62	0.009	2.987 [0.746, 5.228]
	Sex	F(1, 282.94) = 15.54	<0.001	−7.121 [−10.977, −3.265] (higher in women)
	Age	F(1, 282.07) = 1.25	0.264	−0.133 [−0.366, 0.101]
	Sex × Time	F(4, 656.23) = 1.22	0.302	n.s.

Model (omnibus): full-model χ^2^ vs. thresholds-only model; not reported for hydration outcomes. For hunger, thirst, and appetite timing (ordinal outcomes), values are odds ratios [95% CI]; OR < 1 indicates lower odds of a higher category. For absolute and relative hydration (continuous outcomes), values are unstandardized B [95% CI] in original units (male vs. female for Sex; per year for Age). n.s., not significant. A Sex × time term not included in the thirst model (see Methods).

Hunger varied significantly over time in the Muslim group (χ^2^(10) = 105.521, *p* < 0.001). Significant effects were: age (χ^2^(1) = 49.609, *p* < 0.001; B = −0.054, with increasing age associated with a lower hunger category), and time point (χ^2^(4) = 47.506, *p* < 0.001), indicating that relative to time point A, hunger was lower at time points B, C, D, and E (B = −0.824, *p* < 0.001; C = −1.027, *p* < 0.001; D = −1.417, *p* < 0.001; E = −0.893, *p* < 0.001). Sex was not significant (χ^2^(1) = 0.599, *p* = 0.439).

Thirst varied significantly over time in the Muslim group (χ^2^(6) = 67.152, *p* < 0.001). Significant effects were age (χ^2^(1) = 6.529, *p* = 0.011; B = −0.020) and time point (χ^2^(4) = 58.186, *p* < 0.001), indicating that relative to time point A, thirst was higher at time point B (B = 0.379, *p* = 0.020) and time point C (B = 0.474, *p* = 0.004), lower at time point D (B = −0.579, *p* < 0.001), and not significantly different at time point E (B = −0.285, *p* = 0.074). Sex was not significant (χ^2^(1) = 2.502, *p* = 0.114). As described in Statistical Analyses, the sex × time interaction term was not retained in this model due to non-convergence in the full specification.

Specific daily peak appetite timing varied significantly over time in the Muslim group (χ^2^(10) = 77.286, *p* < 0.001). Significant effects were age (χ^2^(1) = 16.381, *p* < 0.001; B = −0.030) and time point (χ^2^(4) = 56.943, *p* < 0.001), indicating that relative to time point A, the specific daily peak appetite timing was delayed at time points B (B = 0.458, *p* = 0.023) and C (B = 0.674, *p* = 0.001), and not significantly different at time points D (B = −0.115, *p* = 0.584) and E (B = −0.361, *p* = 0.082), whereas sex (χ^2^(1) = 0.007, *p* = 0.935) and the sex × time interaction (χ^2^(4) = 4.202, *p* = 0.379) were not significant.

In the linear mixed models, time was a significant predictor of both absolute hydration (F(4, 661.52) = 5.80, *p* < 0.001) and relative hydration (F(4, 656.22) = 5.02, *p* = 0.001) in the Muslim group. For absolute hydration, values were higher at time point E (4 weeks post-Ramadan, B = 0.194, *p* = 0.012) relative to time point A (week before Ramadan), whereas time points B (B = −0.068, *p* = 0.348), C (B = 0.024, *p* = 0.753), and D (B = 0.037, *p* = 0.637) were not significantly different from time point A. For relative hydration, values were also higher at time point E (B = 2.99, *p* = 0.009) relative to time point A, whereas time points B (B = −0.733, *p* = 0.493), C (B = 0.391, *p* = 0.733), and D (B = 0.426, *p* = 0.711) were not significantly different from time point A. Sex was a significant predictor of relative hydration (F(1,282.94) = 15.544, *p* < 0.001), indicating a sex effect, with higher relative hydration in women than men, while age, and the sex × time interaction were not significant predictors of relative hydration (age: F(1,282.07) = 1.25, *p* = 0.264; sex × time: F(4,656.23) = 1.22, *p* = 0.302). Sex, age, and the sex × time interaction were not significant predictors of absolute hydration (sex: F(1,282.035) = 1.508, *p* = 0.221; age: F(1,281.09) = 1.47, *p* = 0.226; sex × time: F(4,661.53) = 1.52, *p* = 0.196).

## Discussion

The current longitudinal survey study among Israeli Muslims found distinct trajectories of hunger and thirst during Ramadan fasting, with hunger suppression contrasting with thirst escalation. These behavioral patterns showed clear sex-related variation, with sex differences most evident in fluid intake and hunger phase transitions. In the phase transition analyses, women exhibited steeper hunger suppression than men, with more abrupt suppression at Ramadan onset and faster rebound during recovery, while post-Ramadan thirst fell below pre-Ramadan levels in both sexes. Additionally, older participants showed earlier appetite timing. Control participants showed no transition-specific changes, despite a small omnibus hunger reduction effect, consistent with fasting-specific adaptation in the Muslim group rather than seasonal effects.

### Dissociated regulation of hunger and thirst

Hunger and thirst followed distinct trajectories across the study period. Hunger declined rapidly at Ramadan onset, remained suppressed through late fasting, and showed incomplete recovery even one month post-Ramadan. In contrast, thirst increased progressively during Ramadan, persisting through the final fasting week, then decreased substantially below baseline during recovery. This dissociation, characterized by sustained hunger suppression alongside incremental thirst elevation despite simultaneous deprivation of both food and water, suggests partially dissociable self-reported trajectories of hunger and thirst during Ramadan fasting and recovery.

Energy and fluid homeostasis differ in their response to short-term deprivation. Hunger is buffered by hepatic glycogen and adipose energy stores, allowing tolerance to short-term energy deficits (31), while thirst signals a more immediate physiological need as dehydration rapidly compromises plasma osmolality and blood volume (3). During Ramadan, hunger was progressively suppressed, whereas thirst remains elevated, which is consistent with a stronger drive to restore fluid balance during dehydration.

The hunger suppression observed here is in line with prior reports of changes in orexigenic signaling, although the specific hormonal drivers remain a subject of scholarly debate. Evidence suggests significant reductions in ghrelin, leptin, and melatonin on day 28 of Ramadan compared with one week prior, with stronger effects in men (15). However, a meta-analysis reported increased ghrelin with no significant changes in leptin, insulin, or gastrin (32), suggesting that hormonal responses may vary across studies and populations.

The Ramadan fast is primarily defined by temporal reorganization of eating/fasting windows across the 24 h cycle rather than caloric restriction, distinguishing it from other forms of intermittent fasting. Ramadan uniquely combines prolonged duration (28–29 days), complete daytime fluid restriction, circadian misalignment, and religious motivation, features not replicated in time-restricted eating or alternate-day fasting protocols. Unlike weight-loss-motivated intermittent fasting, where hunger persists (33), Ramadan fasting is embedded in a context of religious obligation, spiritual meaning, and collective participation, alongside a month-long shift in eating and drinking routines (34). The sustained hunger suppression one month post-Ramadan, when meal timing is unrestricted, suggests effects persisting beyond fasting cessation. This prolonged adaptation is notable given the modest ~3.5% weight loss among Muslim participants, which would typically be expected to elicit compensatory increases in weight (35).

### Temporal dynamics across fasting phases

Peak daily hunger level exhibited a stepwise reduction across two major transitions: an initial decline at Ramadan onset and a second decrease after unrestricted food access was restored. The lowest peak daily hunger level occurred one week post-Ramadan, when 30% reported moderate to strong hunger, lower than during late fasting (42%), suggesting that the fasting-related reduction in hunger sensation did not reverse immediately after fasting ended. Between one week and one month post-Ramadan, peak daily hunger level increased but remained significantly below baseline (A → E: Z = −6.023, *p* < 0.001), indicating sustained suppression at least one month beyond fasting cessation.

Consistent with sustained hunger suppression, participants reported modest weight loss, exceeding the ~1 kg reported in meta-analysis (36), but aligning with the 1.6 kg reduction in the LORANS cohort (37), losses typically regained within weeks in healthy populations (35). Pooled evidence indicates slight energy intake reduction during Ramadan (−142.5 kcal/day) with larger water intake reductions, though with substantial heterogeneity (38). Cardiometabolic markers show modest, transient improvements including increased HDL, reduced LDL and total cholesterol, lower systolic blood pressure, and reduced fasting glucose (39, 40), with metabolomic data suggesting potential favorable shifts in chronic disease–related pathways (14). Interpretation remains limited by protocol diversity, sex bias (male overrepresentation) and variable study quality.

Appetite timing showed more rapid recovery dynamics. Peak appetite shifted to later hours during Ramadan, reflecting alignment with the delayed eating window and extended wakefulness characteristic of Ramadan circadian reorganization. Daily peak appetite timing returned to an earlier daily phase at early recovery and remained stable through late recovery, indicating that circadian appetite timing adjusted more quickly than hunger intensity, which required longer recalibration. Meal timing during Ramadan can influence performance, as demonstrated in adolescent female athletes who maintained better cognitive performance with later pre-dawn meal timing (41). Recent work on shifted sleep–wake schedules emphasizes the interdependence with daytime eating patterns (42) with major implications for cardiometabolic health (43, 44).

Unlike hunger, which decreased at Ramadan onset and remained below baseline throughout recovery, thirst followed a biphasic trajectory, rising progressively during fasting, then decreasing below pre-Ramadan baseline during recovery. Despite elevated thirst, fluid intake remained stable during Ramadan through compensatory nighttime drinking, maintained somewhat below recommended levels (EFSA: 2.5–3.7 L/day men, 2.0–2.7 L/day women) (45, 46), particularly among men. Strategic overnight fluid intake likely supported fluid balance during Ramadan, consistent with evidence that 2–3 L overnight can prevent elevated serum creatinine and urea (47, 48). Post-Ramadan, fluid intake increased markedly, exceeding baseline levels, paralleling findings from Finch et al. (49). This rebound, not seen in controls, whose absolute and relative hydration showed no significant time effect (Supplementary material SM-4), confirms that these effects were beyond seasonal changes. The persistent elevation suggests continued compensatory drinking, with intake approaching recommended levels. Reports of heat-related illness during Ramadan highlight risks, particularly as global warming increases overlap between summer fasting and heat exposure (29).

These temporal patterns reveal that recovery is not simply a reversal of adaptation: daily peak appetite timing returned to baseline more quickly than daily peak hunger level, thirst fell below baseline during recovery, whereas fluid intake increased above baseline. This asynchronous recalibration highlights the complexity of regulatory responses following prolonged fasting. The control group’s small omnibus hunger effect (Friedman: χ^2^(4, 75) = 15.41, *p* = 0.004, η^2^ = 0.051) was not accompanied by a significant time effect in the corresponding ordinal model (χ^2^(4) = 7.64, *p* = 0.106), significant transition-specific changes, or corresponding thirst or hydration changes, suggesting non-fasting influences, potentially environmental or seasonal factors. The etiology remains unclear.

Meta-analytic evidence finds no differential hunger effect versus caloric restriction (33), yet Ramadan cannot be equated with standard intermittent fasting protocols because it uniquely combines a dramatic shift in eating timing to evening and nighttime, complete daytime fluid restriction, sleep disruption, and religious motivation. Despite the importance of temporally structured biomarker sampling across before-early-late Ramadan and early-late recovery phases, few studies implement these requirements, limiting mechanistic interpretation. Finch et al. (49) provided an early longitudinal assessment of daily hunger and drinking patterns during Ramadan, though in a small sample (*n* = 41) with brief assessments. The present study builds on this methodological approach as one of the first controlled multi-timepoint longitudinal studies to examine self-reported hunger, thirst, appetite timing, and fluid-intake behavior across Ramadan and recovery, with a larger sample (*n* = 287), a non-fasting, ethnically comparable control group, and greater temporal resolution and statistical power for sex-stratified analyses than prior work in this area.

### Sex- and age-specific adaptations

Women demonstrated greater overall hunger suppression than men, as evidenced by a larger Friedman effect (women: χ^2^(4,162) = 63.20, *p* < 0.001, η^2^ = 0.098; men: χ^2^(4,119) = 18.17, *p* = 0.001, η^2^ = 0.038), In women, significant pairwise transitions occurred at A → B and D → E, whereas men showed no significant pairwise transitions. Paradoxically, at one month post-Ramadan, women showed higher frequencies of moderate to strong hunger, indicating a faster post-Ramadan increase in hunger than men, though remaining below pre-Ramadan levels. These findings contrast Finch et al. (49), who found women experienced higher absolute hunger than men during early Ramadan in a small British Muslim sample (*n* = 41), attributing this to differential food cue exposure through meal preparation. Our opposite finding suggests factors beyond food cue exposure drive sex differences. Methodological differences may also contribute, as Finch et al. assessed only two timepoints, whereas our five-timepoint design captured dynamic trajectories revealing more abrupt hunger transitions in women, reflecting steeper suppression at onset and faster increase during recovery (49). Thirst dynamics showed sex-independent trajectories overall. Post-Ramadan fluid intake diverged: men showed immediate increases at one week post-Ramadan, while women showed gradual increases across both recovery phases.

Sex differences in substrate metabolism may help explain the observed patterns. Men and women exhibit dimorphism in glucose and lipid metabolism during fasting (18), and sex-specific brain responses to hunger and satiety signals (19). The stronger hormonal effects observed in men by Al-Rawi et al. (15), including greater ghrelin and leptin reductions may paradoxically result in less pronounced subjective hunger suppression if women’s brain circuits are more sensitive to remaining orexigenic signals. Sex-specific patterns during Ramadan extend beyond homeostatic outcomes, as female CiRAM participants exhibited a transient elevation in psychological distress during fasting alongside a more pronounced decline in subjective quality of life (50).

Daily fluid intake normalized to body showed a consistent sex difference: Women consumed significantly more fluid per kg body weight than men across all time points, with a similar sex difference seen in the control sample at several time points (Supplementary material SM-3), in line with prior population studies showing higher 24-h urine osmolality in men (51). Muslim men in our sample reported consuming less than recommended fluid volumes throughout the study, raising concerns about chronic dehydration risk, particularly when Ramadan occurs during summer.

Although our sample is predominantly young (mean age 26.9 ± 6.77 years), older age was associated with earlier daily peak appetite onset in Muslim participants (ordinal generalized linear model, age effect: χ^2^(1) = 16.38, *p* < 0.001; B = −0.030). This pattern is consistent with age-dependent changes in chronotype (52). Beyond biological aging, the earlier appetite peaks may also reflect long-term entrainment to repeated daily routines. Decades of Ramadan observance and year-round early-morning practices, such as the Fajr prayer, may be associated with earlier appetite timing through greater circadian regularity (53). These findings contrast with a longitudinal UK study of 2,945 participants, in which older age was associated with later meal times and narrower eating windows (54). Such a discrepancy suggests that age-related shifts in meal timing are modulated not only by biology but also by cultural and religious zeitgebers.

### Implications for practice and policy

Current findings have direct relevance for healthcare providers counselling Ramadan-observing patients. Hydration management emerges as a critical concern. Strategic nighttime fluid intake (2–3 L between sunset and pre-dawn meals) is recommended, especially during summer months with longer fasting windows and higher ambient temperatures. Evidence demonstrates this approach maintains kidney function during Ramadan (48), though individual variation based on body size, activity level, and environmental conditions requires personalized assessment.

Sex-specific counselling should recognize differential patterns across the Ramadan period: in the phase-transition analyses, women demonstrated greater hunger suppression, yet showed faster post-Ramadan hunger resurgence. Men showed a more gradual attenuation in both hunger and thirst, with consistently lower normalized fluid intake throughout the study period, warranting attention to hydration, particularly in male fasters. Age-related patterns also warrant consideration: older adults showed lower overall hunger intensity and earlier appetite timing, which may support adherence to pre-dawn meals and facilitate adequate nutrient intake. Age-related decline in thirst sensitivity may further compromise hydration adequacy, as older individuals may not perceive thirst despite physiological need (16, 17).

The modest weight loss reported during Ramadan (2–3 kg) is consistent with healthy, reversible changes in non-obese populations (55), although monitoring is appropriate for individuals with low baseline BMI. Weight management implications should be interpreted in light of the transient nature of Ramadan-related weight loss, which is usually regained within weeks after fasting ceases. Those concerned with excessive weight loss should be counselled about gradual hunger return and the possible need for increased dietary intake post-Ramadan.

These findings also inform clinical fasting protocols and time-restricted eating interventions beyond Ramadan. The demonstration that thirst, but not hunger, resists sustained suppression may inform behavioral interventions distinguishing these drives and their amenability to voluntary override.

### Limitations

This study, part of the CiRAM project, focused on appetite and thirst behavior and relied on self-reports without comprehensive nutritional assessment and biological measurements. Hunger, thirst, and appetite timing were assessed using single-item self-report measures rather than validated multi-item scales. The survey instrument had not undergone formal pilot testing or face-validity assessment prior to administration. Fluid intake was estimated in 250 mL cup equivalents, which excludes water from food sources and likely underestimates total water intake.

The control group was ethnically, culturally, and geographically comparable to the Muslim sample but not individually or frequency matched, and the groups differed significantly in some socio-demographic parameters. The control group was collected entirely in 2023, whereas the Muslim sample spanned 2021–2023. A sensitivity analysis restricted to the 2023 Muslim subsample showed the age, marital status, and education differences were no longer significant, indicating they largely reflected this multi-year collection rather than a stable group distinction. Area and religious lifestyle differences persisted, indicating genuine group differences independent of year (Supplementary material SM-5). Participant retention also varied by year (Supplementary material SM-1), and as a complete-case analysis, attrition-related bias cannot be ruled out.

The sample was drawn through convenience sampling, likely producing a more health-conscious group than the general population, and was relatively young, Israeli, Muslim, and based in a Middle Eastern climate, with data collected during spring Ramadans (fasting windows of roughly 13–15.5 h). These characteristics may limit generalizability to other Muslim populations fasting under different conditions. Sex was assessed as a binary self-reported variable (male or female) and does not capture gender diversity or non-binary identities. Some participants reported fasting exemptions, including menstruation, and were retained within their cohorts. Weight change was reported at one month post-Ramadan by a subsample (62–65% response rate).

### Future directions

What actually drives hunger’s suppression, whether ghrelin and leptin sensitivity, the brain’s reward response to food cues, or a shift in metabolic set-point, is still an open question, one that neuroimaging (56) and hormonal sampling before and after Ramadan (15) might help answer. Thirst, by contrast, intensified over the course of the fast, suggesting a more tightly regulated physiological signal. Whether this reflects osmotic or volumetric regulation or altered vasopressin dynamics (17) has not been tested in the context of prolonged voluntary fasting.

Longer follow-up, ideally three to six months, and eventually across consecutive Ramadan years, would clarify whether the incomplete one-month recovery is temporary or lasting. This sample also reflects one population under one set of conditions. Ramadan-observing Muslims fast worldwide under vastly different day lengths and climates, and clinicians currently have little evidence to draw on. This gap includes pregnant or lactating women, older adults, and those with chronic disease.

## Conclusion

Ramadan fasting produced partially dissociable self-reported trajectories of hunger and thirst, with progressive hunger suppression contrasting with incremental thirst escalation throughout fasting. In the phase transition analyses, women’s hunger dropped at Ramadan’s onset and rose again during recovery, a pattern not seen in men. Recovery trajectories were asynchronous: appetite timing returned to pre-Ramadan timing within one week while hunger remained suppressed and thirst decreased below pre-Ramadan baseline levels one month post-Ramadan. Control participants did not show comparable transition-specific changes, suggesting these patterns are unlikely to be explained solely by seasonal variation.

As an ecological model unavailable in controlled settings for ethical reasons, Ramadan fasting provides unique insights into dissociable changes in self-reported appetite and thirst, circadian reorganization of eating patterns, and behavioral changes extending beyond the fasting period itself. Healthcare providers counselling patients during fasting periods should consider that older participants showed lower hunger and earlier appetite timing, while women consumed more fluid per kg body weight than men, suggesting that nutritional and hydration guidance may need to be individualized rather than uniformly applied. This work highlights the temporal organization of eating and drinking as a distinct dimension of chrono-nutrition, closely linked to changes in daily hunger, thirst, and appetite timing across the Ramadan fasting-recovery cycle.

## Data Availability

The datasets presented in this article are not readily available because all information necessary for understanding the study and its findings is included in this manuscript and its Supplementary materials. The datasets generated and analyzed during the current study are available from the corresponding author upon reasonable request, subject to participant confidentiality protections and ethical approval restrictions. Requests to access the datasets should be directed to FC, Chenfl@ariel.ac.il; KM, maria.korman@ariel.ac.il.
