# Supplementary material for: When hunger and thirst part ways: a longitudinal comparison of men and women across the Ramadan fasting-recovery cycle, with non-fasting controls
Source: Front Nutr. 2026 Sep 18;13:1859780. doi: 10.3389/fnut.2026.1859780 (PMC13630704; doi:10.3389/fnut.2026.1859780)
Supplement: Supplementary file 1 [file Supplementary_file_1.DOCX]

**Supplementary Materials**

**Supplementary Material SM-1: Study participation and Attrition**

This segment contains a detailed description of the number of participants per year, attrition, and compliance rates.

At time point A (cumulatively in study years 2021-2023), 417 Muslim and 101 non-Muslim individuals were enrolled in the study. Table SM-1 depicts the attrition and exclusion from the study, by participation year, in the study (Muslim) and control (non-Muslim) groups.

Attrition and data quality related exclusions from the original number of enrolled participants at Point A (417 individuals) are shown in Table SM-1, demonstrating participant counts, percent of females by year and time point. The final study sample was comprised of 287 participants that completed all five surveys at the five study time points (A-E), whose answers were verified as valid data.

In 2021, 131 participants (48% female) enrolled at baseline (Point A), with minimal attrition during data collection. Following data quality checks to exclude missing, partial, or corrupted data, the final analytic sample comprised 72 participants (48.6% female), representing a 45.0% exclusion rate from baseline enrollment and a 40.0% exclusion rate among study completers. Female representation remained stable throughout data collection and post-quality control procedures. In 2022, 156 participants (58% female) enrolled at baseline, and after quality checks, 126 participants (57.1% female) remained in the final analytic sample, corresponding to a 19.2% exclusion rate. In 2023, 130 participants (61% female) enrolled at baseline, and after quality checks, 89 participants (67.4% female) remained, corresponding to a 31.5% exclusion rate.

Overall, female representation increased slightly across years (from 48% in 2021 to 61% in 2023).

**Table SM-1:** Participant Attrition Year and Time Point, counts in absolute numbers through Points A-E, % - females and Final – after quality checks that lead to excluding missing, partial or corrupted data.

| **Year** | **Point A (baseline)** | **Point B (adaptation)** | **Point C (stabilization)** | **Point D (recovery I)** | **Point E (recovery II)** | **Final n, after data quality check** |
| --- | --- | --- | --- | --- | --- | --- |
| 2021 | 131 (48%) | 125 (48%) | 120 (47.5%) | 120 (47.5%) | 120 (47.5%) | 72 (48.6%) |
| 2022 | 156 (58%) | 146 (58%) | 140 (58%) | 138 (58%) | 138 (58%) | 126 (57.1%) |
| 2023 | 130 (61%) | 122 (61%) | 114 (61%) | 112 (61%) | 94 (61%) | 89  (67.4%) |
| 2023  (non-Muslim control | 101 (62%) | 95  (63%) | 88  (60%) | 88  (60%) | 88  (60%) | 76  (60.5%) |

**Data Quality Improvements**

In the first data collection year (2021), approximately 42% of survey items were flagged as invalid due to missing, partial, or corrupted responses. In subsequent years (2022 and 2023), this rate decreased to 6-9%, largely due to the implementation of structured reminders at each time point. These reminders emphasized accuracy (e.g., “long surveys make you prone to input errors”) and informed participants that the experimenter might contact them within 24 hours of submission to review ambiguous responses. This procedural enhancement substantially improved data integrity and reduced exclusion rates.

**Supplementary Material SM-2: The direction of change in peak appetite timing**

Since daily timing was represented by 6 possible answers, including one answer describing a steady appetite throughout the day, we conducted an analysis of the direction of change for each participant by comparing the trend in their appetite timing between study phases (see methods). For each paired comparison representing a transition between study phases, approximately 13-14.5% of participants within the fasting Muslim sample had reported having a steady appetite throughout the day in one or two of the time points in the paired comparison. In pair A-B, while transitioning into the Ramadan Fast, nearly 40% of Muslim participants reported a later timing for the daily peak hunger, while just under 30% reported the same timing (no change), and close to 18% reported an earlier timing. During Ramadan (Pair B-C), most Muslim participants (44.6%) reported the same timing (no change) for daily peak hunger, ~21% reported an earlier timing and ~22% reported a later timing for peak daily appetite. Emerging from the fast to the following week (C-D) shifted the distribution towards earlier peak daily appetite among Muslims (38.3%), with ~28% reporting the same daily timing (no change) and ~20% reporting a later peak daily timing of hunger. Progression from the end of the fast into the first week of recovery (C-D) is the only phase transition which displayed a statistically significant difference between the Muslim and control groups as indicated by a Pearson’s χ^2^ test: χ²(3, N = 363) = 18.17, p < .001. Muslim participants were more often in the “Earlier” category (ASR = 3.9, p < .001) and less often in “No change” (ASR = -2.8, p = .005) compared with control participants. When recovering from 1 to 4 weeks post Ramadan (pair D-E), 39% of participants reported the same timing of peak daily hunger (no change), 24% reported earlier, and 23.3% reported later peak daily hunger. No significant difference was observed between male and female Muslims in all paired comparisons of phase transitions, as indicated by a Pearson’s χ^2^ test: χ²(3, 287): A–B = 2.140, p = .544; B–C = 2.814, p = .421; C–D = 2.407, p = .492; D–E = 5.609, p = .132 (Figure 5, panels a and b).

The control group displayed no significant changes across the 5 points (A–E) (χ² (4, 75) = 4.278, p = .370). Additionally, the between-group comparisons showed that the pair C–D (early recovery) is the only phase transition with a statistically significant difference between the Muslim and control groups as indicated by a Pearson’s χ² test: χ²(3, 363) = 18.17, p < .001. Muslim participants were more often in the “Earlier” category (ASR = 3.9, p < .001) and less often in “No change” (ASR = -2.8, p = .005) compared with control participants.

**Supplementary Material SM-3:**

**Mean ± St. Dev. of daily fluid intake** in absolute (Liters/day) and normalized (Liters/Kg /day) values, in the Total Control, Male and female Control populations. Ramadan time points (B & C) appear in light green.

|  | **Liters / day**  **Mean±St.Dev** | | | | | **mL / Kg / day**  **Mean±St.Dev** | | | | |
| --- | --- | --- | --- | --- | --- | --- | --- | --- | --- | --- |
|  | **Total Control** | **Men** | **Women** | **Sex comparison**  **(Mann-Whitney U test)** | | **Total Control** | **Men** | **Women** | **Sex comparison**  **(Mann-Whitney U test)** | |
| **n** | **75-76** | **28** | **47-48** | **z** | **p** | **75-76** | **28** | **47-48** | **z** | **p** |
| **A** | 2.69 ± 1.13 | 2.59 ± 1.14 | 2.75 ± 1.13 | -0.667 | 0.505 | 40.90 ± 20.07 | 32.51 ± 15.77 | **45.80 ± 20.81** | **-2.832** | **0.005** |
| **B** | 2.83 ± 1.00 | 2.95 ± 0.94 | 2.76 ± 1.03 | -0.897 | 0.369 | 42.26 ± 16.98 | 36.64 ± 13.21 | **45.60 ± 18.18** | **-2.032** | **0.042** |
| **C** | 2.90 ± 1.06 | 2.82 ± 0.89 | 2.95 ± 1.15 | -0.450 | 0.653 | 44.01 ± 19.72 | 35.09 ± 12.16 | **49.21 ± 21.46** | **-3.177** | **0.001** |
| **D** | 2.73 ± 1.03 | 2.88 ± 1.05 | 2.64 ± 1.01 | -0.839 | 0.401 | 41.11 ± 17.33 | 36.16 ± 14.63 | 44.00 ± 18.25 | -1.922 | 0.055 |
| **E** | 2.92 ± 1.05 | 2.87 ± 0.84 | 2.95 ± 1.16 | -0.211 | 0.833 | 44.36 ± 19.41 | 35.86 ± 12.20 | **49.32 ± 21.16** | **-2.730** | **0.006** |

**Figure SM-3: Reported Daily Fluid Intake,** in Liters per day (a) and in daily mL, normalized to body weight (mL/Kg/day) (b). Boxplots displayed for each study phase (A-E), shown **for Muslim men & women**. Significant planned phase transitions (Bonferroni corrected, ɑ = 0.0125), are indicated by an asterisk (*). Ramadan time points (B & C) appear in light green.

|   *  * |   **Women**  **Men**  *  * |
| --- | --- |
| **a. Liters / Day**  **Women**  **Men** | **b. mL / Kg / Day** |

**Supplementary Material SM-4:**

**Results from the mixed models:**

In the control group, hunger varied significantly over time in the omnibus model (χ²(10) = 28.164, p = .002). Relative to time point A, hunger was significantly lower at time points D (B = -1.001, p = .012) and E (B = -0.892, p = .026), but not at time points B (B = -0.361, p = .382) or C (B = -0.573, p = .160). Age was significantly associated with hunger, indicating that older age was associated with lower hunger (χ²(1) = 13.753, p < .001), whereas sex (χ²(1) = 2.641, p = .104) and the sex × time interaction (χ²(4) = 2.685, p = .612) were not significant.

Thirst did not vary significantly over time in the control group (χ²(6) = 8.477, p = .205). Relative to time point A, thirst was significantly lower only at time point E (B = -0.675, p = .038); it was not significantly different at time points B (B = -0.164, p = .615), C (B = -0.344, p = .292), or D (B = -0.458, p = .153). Sex (χ²(1) = 1.989, p = .158) and age (χ²(1) = 1.121, p = .290) were also not significant.

Specific daily appetite timing did not vary significantly over time in the control group (χ²(10) = 11.949, p = .288). Relative to time point A, appetite timing was significantly earlier at time point B (B = -0.819, p = .033) and time point E (B = -0.913, p = .020), but not at time points C (B = -0.659, p = .090) or D (B = -0.484, p = .198). Sex (χ²(1) = 0.378, p = .539), age (χ²(1) = 2.365, p = .124), and the sex × time interaction (χ²(4) = 3.229, p = .520) were also not significant.

In the linear mixed model for absolute hydration, time was not a significant predictor of absolute hydration in the control group (F(4, 148.213) = 1.691, p = .155). Sex (F(1, 71.223) = 0.000, p = .999), age (F(1, 72.324) = 0.022, p = .882), and the sex × time interaction (F(4, 148.214) = 2.016, p = .095) were also not significant.

In the relative hydration model, sex was significant, indicating higher relative hydration in women than men (F(1, 70.944) = 8.643, p = .004). Time was not a significant predictor of relative hydration in the control group (F(4, 143.878) = 1.278, p = .281). Age (F(1, 71.904) = 1.056, p = .308) and the sex × time interaction (F(4, 143.878) = 1.730, p = .146) were also not significant.

**Table SM-4.** Ordinal generalized linear model and linear mixed-model results for daily peak hunger, thirst, appetite timing, and hydration in the **non-fasting control group**.

| **Outcome** | **Predictor** | **Statistic** | **p** | **OR / B [95% CI]** |
| --- | --- | --- | --- | --- |
| **Peak Daily Hunger level** | Model (omnibus) | χ²(10) = 28.16 | .002 | — |
|  | Time  (with A as Baseline) | χ²(4) = 7.64 | .106 | — |
|  | *B vs. A* | χ²(1) = 0.77 | .382 | 0.70 [0.31, 1.56] |
|  | C vs. A | χ²(1) = 1.97 | .160 | 0.56 [0.25, 1.25] |
|  | D vs. A | χ²(1) = 6.26 | .012 | 0.37 [0.17, 0.81] |
|  | E vs. A | χ²(1) = 4.94 | .026 | 0.41 [0.19, 0.90] |
|  | Sex | χ²(1) = 2.64 | .104 | 0.63 [0.24, 1.62] |
|  | Age | χ²(1) = 13.75 | <.001 | 0.94 [0.91, 0.97] |
|  | Sex × Time | χ²(4) = 2.69 | .612 | n.s. |
| **Peak Daily Thirst level** | Model (omnibus) | χ²(6) = 8.48 | .205 | — |
|  | Time  (with A as Baseline) | χ²(4) = 5.22 | .266 | — |
|  | B vs. A | χ²(1) = 0.25 | .615 | 0.85 [0.45, 1.61] |
|  | C vs. A | χ²(1) = 1.11 | .292 | 0.71 [0.37, 1.34] |
|  | D vs. A | χ²(1) = 2.04 | .153 | 0.63 [0.34, 1.19] |
|  | E vs. A | χ²(1) = 4.30 | .038 | 0.51 [0.27, 0.96] |
|  | Sexa | χ²(1) = 1.99 | .158 | 0.74 [0.49, 1.12] |
|  | Age | χ²(1) = 1.12 | .290 | 0.98 [0.95, 1.01] |
| **Specific**  **Peak Daily Appetite timing** | Model (omnibus) | χ²(10) = 11.95 | .288 | — |
|  | Time  (with A as Baseline) | χ²(4) = 5.10 | .277 | — |
|  | B vs. A | χ²(1) = 4.55 | .033 | 0.44 [0.21, 0.94] |
|  | C vs. A | χ²(1) = 2.87 | .090 | 0.52 [0.24, 1.11] |
|  | D vs. A | χ²(1) = 1.66 | .198 | 0.62 [0.30, 1.29] |
|  | E vs. A | χ²(1) = 5.45 | .020 | 0.40 [0.19, 0.86] |
|  | Sex | χ²(1) = 0.38 | .539 | 0.66 [0.27, 1.65] |
|  | Age | χ²(1) = 2.37 | .124 | 0.98 [0.95, 1.01] |
|  | Sex × Time | χ²(4) = 3.23 | .520 | n.s. |
| **Absolute hydration** | Time  (with A as Baseline) | F(4, 148.21) = 1.69 | .155 | — |
|  | B vs. A | t(205.02) = 0.37 | .715 | 0.047 [−0.205, 0.298] |
|  | C vs. A | t(229.71) = 0.97 | .332 | 0.134 [−0.137, 0.405] |
|  | D vs. A | t(176.96) = −1.36 | .176 | −0.183 [−0.450, 0.083] |
|  | E vs. A | t(169.28) = 1.10 | .274 | 0.150 [−0.120, 0.419] |
|  | Sex | F(1, 71.22) = 0.00 | .999 | −0.232 [−0.768, 0.303] |
|  | Age | F(1, 72.32) = 0.02 | .882 | −0.002 [−0.036, 0.031] |
|  | Sex × Time | F(4, 148.21) = 2.02 | .095 | n.s. |
| **Relative hydration** | Time  (with A as Baseline) | F(4, 143.88) = 1.28 | .281 | — |
|  | B vs. A | t(202.76) = 0.25 | .803 | 0.516 [−3.554, 4.586] |
|  | C vs. A | t(227.68) = 1.02 | .307 | 2.292 [−2.117, 6.701] |
|  | D vs. A | t(170.91) = −1.35 | .178 | −2.970 [−7.307, 1.368] |
|  | E vs. A | t(162.19) = 1.24 | .219 | 2.742 [−1.642, 7.125] |
|  | Sex | F(1, 70.94) = 8.64 | .004 | −14.284 [−23.369, −5.198]  (higher in women) |
|  | Age | F(1, 71.90) = 1.06 | .308 | −0.295 [−0.869, 0.278] |
|  | Sex × Time | F(4, 143.88) = 1.73 | .146 | n.s. |

*Note. Model (omnibus): full-model χ² vs. thresholds-only model; not reported for hydration outcomes. For hunger, thirst, and appetite timing (ordinal outcomes), values are odds ratios [95% CI]; OR < 1 indicates lower odds of a higher category. For absolute and relative hydration (continuous outcomes), values are unstandardized B [95% CI] in original units (male vs. female for Sex; per year for Age). n.s., not significant. A Sex × time term not included in the thirst model (see Methods).*

**Supplementary Material SM-5:**

**Year-Restricted Sensitivity Analysis**

The control group was collected entirely in 2023, whereas the Muslim fasting sample was collected across 2021–2023. To evaluate whether the demographic differences reported between groups (Table 1) reflect a genuine group distinction or an artifact of this differential year coverage, the five significant Muslim-vs-Control demographic comparisons were re-run restricted to participants enrolled in 2023 only (Muslim n = 89; Control n = 76). Results are compared against the original full-sample statistics below.

| **Variable** | **Full sample (n = 363)** | **2023-only subsample (n = 165)** | **Result** |
| --- | --- | --- | --- |
| Age | Mann-Whitney U, Z = -3.70, p < .001 | Z = -0.39, p = .694 | No longer significant |
| Marital status | χ²(1) = 16.22, p < .001, V = .21 | χ²(1) = 2.91, p = .088, V = .13 | No longer significant |
| Education | χ²(3) = 13.49, p = .004, V = .19 | χ²(3) = 3.17, p = .367, V = .14 | No longer significant |
| Geographic area | χ²(4) = 39.50, p < .001 | χ²(3) = 8.58, p = .035, V = .23 | Persisted (attenuated) |
| Religious lifestyle | χ²(3) = 61.12, p < .001, V = .41 | χ²(3) = 21.79, p < .001, V = .36 | Persisted |

*Note. χ² = Pearson chi-square; V = Cramér's V. Age was tested with the Mann-Whitney U test given its continuous, non-normally distributed structure. Full-sample statistics are as reported in Table 1 and the main text. The 2023-only subsample restricts the Muslim group to participants enrolled during the 2023 Ramadan period, matching the Control group's single year of collection.*
